# A Traditional Gum Exudate From *Pistacia atlantica* Ameliorates Etha‐Nol‐Mediated Gastric Ulcer in Rats: Possible Molecular Mechanisms

**DOI:** 10.1002/fsn3.70049

**Published:** 2025-03-02

**Authors:** Talal Salem Al‐Qaisi, Ahmed A. J. Jabbar, Mohammed M. Hussein M. Raouf, Najat Jabbar Ahmed Berwary, Qosay Al‐Balas, Parween Abdul‐Samad Ismail, Muzhda Haydar Saber, Ramzi A. Mothana, Abdullah R. Alanzi, Mahmood Ameen Abdulla, Rawaz Rizgar Hassan, Musher Ismael Saleh, Sidgi Hasson

**Affiliations:** ^1^ Department of Biomedical Sciences, College of Health Sciences Abu Dhabi University Abu Dhabi UAE; ^2^ Department of Medical Laboratory Sciences, Faculty of Allied Medical Sciences Al‐Ahliyya Amman University Amman Jordan; ^3^ Department of Medical Laboratory Technology Erbil Technical Health and Medical College, Erbil Polytechnic University Erbil Iraq; ^4^ Department of Biomedical Sciences, College of Science Cihan University‐Erbil Erbil Iraq; ^5^ Department of Medicinal Chemistry and Pharmacognosy, Faculty of Pharmacy Jordan University of Science and Technology Irbid Jordan; ^6^ Department of Chemistry, College of Education Salahaddin University Erbil Iraq; ^7^ Department of Nursing Lebanese French University Erbil Kurdistan Region Iraq; ^8^ Department of Pharmacognosy College of Pharmacy, King Saud University Riyadh Saudi Arabia; ^9^ Department of Medical Analysis, Faculty of Applied Science Tishk International University Erbil Iraq; ^10^ Department of Medical Laboratory Science, College of Science Knowledge University Erbil Iraq; ^11^ Department of Chemistry, Faculty of Science and Health Koya University Erbil Iraq; ^12^ School of Pharmacy and Biomolecular Sciences Liverpool John Moores University Liverpool UK

**Keywords:** antioxidant, gastric ulcer, histopathology, inflammatory cytokine, *Pistacia atlantica*, toxicity

## Abstract

*Pistacia atlantica* (Bene) is a native Mediterranean plant that exudates a resinous therapeutic oleoresin gum used for many inflammatory‐related diseases. Therefore, the present study evaluates the acute toxicity and the gastroprotective effects of *Pistacia atlantica* gum (PAG) on ethanol‐mediated gastric ulcers in rats. Sprague Dawley rats (30) were placed in 5 cages: Group A received 10% tween 20; Group B, ulcer control, received 10% tween 20; reference rats (C, received 20 mg/kg omeprazole), groups D and E received 250 and 500 mg/kg of PAG, respectively. After 60 min, Groups B–E rats received absolute ethanol (5 mL/kg). The acute toxicity results showed a lack of any physiological alterations in rats supplemented with up to 5 g/kg of PAG. In the gastroprotective trial, the ulcer controls exhibited extensive gastric mucosal injuries, reduced stomach mucus secretion, a highly acidic stomach, and increased lesion areas. Ethanol ingestion caused significant inflammatory cell infiltration and oxidative stress indicators in their gastric tissues. The ulcer controls revealed reduced HSP 70, elevated Bax protein expressions, and lowered antioxidant enzymes and up‐regulated MDA contents. PAG treatment restored these negative effects of ethanol, which could be because of its terpenoids, phenolics, and flavonoids potentials that positively modulated the gastric defense barriers (mucopolysaccharides), gastric antioxidants, mucus secretion, and significantly increased gastric pH and reduced inflammatory cytokines (TNF‐α and IL‐6). The gastro‐prophylactic potentials are validated by its modulatory actions of gastric defense mechanisms, providing scientific evidence for future biochemical characterizations.

## Graphical abstract

*Pistacia atlantica* gum (PAG) is a native Mediterranean plant that exudates a resinous therapeutic oleoresin gum used for many inflammatory‐related diseases—PAG has shown significant anti‐ulcer potential against ethanol‐mediated ulcers in rats. PAG supplementation restored gastric defense barriers (mucopolysaccharides), mucus secretion, and significantly increased gastric pH, up‐regulated SOD, CAT, PGE2, and IL‐10, while reducing inflammatory cytokines (TNF‐α and IL‐6). PAG gastroprotection could be linked to its phytochemical potential in modulating antioxidants, Bax, and HSP 70 proteins.

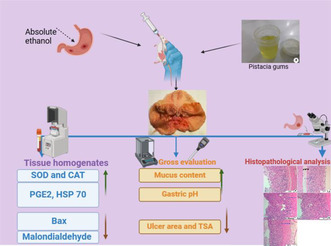

## 1 Introduction

Peptic ulcer disease (PUD) is a well‐known disease resulting from disturbed homeostasis between gastric mucosal defense and destructive factors that consequently lead to gastrointestinal injury with increased rates of morbidity and mortality in the last several decades. Lately, scientists labeled PUD as the new plague of the 21st century because of the elevated incidence rate worldwide (Malfertheiner and Schulz 2020). As a deleterious painful disease, Peptic ulcer has been diagnosed in more than four million U.S. citizens, based on a newly released statistic, and occurs in 1–10 cases during their lifetime (Beiranvand 2022).

Until now, the exact physiologic pathway of PUD is not fully understood. The latest hypothesis declares that several interactions, along with different factors, including genetics, the host immune system, and environmental factors, interfere with intestinal homeostasis, progressing into dysregulated inflammatory reactions of the gastrointestinal tract (GI) (Gilani et al. 2022). Gastric ulcers are one of the outcomes of the mentioned interference with intestinal homeostasis, which is commonly found in the stomach and proximal duodenum based on previously published study trials (Jabbar, Mothana, et al. 2023; Mansour et al. 2022). Studies also introduced gastric ulcers as a result of disrupting the gastric defense system, including mucus content, endogenous defense enzymes, mucosal membranes, and cell regeneration, thereby reducing the biological actions of GI (Ahmed et al. 2022).

Alcohol has been known to stimulate gastric lesions that lead to the reduction of the gastric defense system, mucosal spreading, and mucus content (Ammar et al. 2022). Consuming alcohol (Ethanol) may lead to necrotic lesion: in the mucosa lining of the stomach by different pathways: direct necrotic lesion initiation (causing reduction of the gastric defensive factors) and decreased release of bicarbonate (Geetha and Anitha 2022). Layers of the gastric mucosa are a well‐known defensive factor against gastrointestinal damage. Gastric Mucosal secretion is known as a gel‐mucous barrier released by different gastric cells that play an important defensive role in preventing gut injuries that may occur from acid and digestive enzymes. Scientists have shown that measuring the gastric mucus content can be a convenient technique to estimate stomach mucosal secretion (Taha et al. 2015).

Natural products have been known as effective suppressors of the pro‐inflammatory pathway, thereby reducing the rate of gastric ulcer formation; however, their application in the production of pharmaceutical and biomedical products is continuously remodeled to obtain maximum efficiency (Jabbar et al. 2022a; Jabbar, Mothana, et al. 2023; Ahmed et al. 2024). Phytochemicals, including terpenoids, alkaloids, and phenols, have been known as chemical contents of plants that play important biological actions as antioxidants by increasing endogenous antioxidant enzymes (CAT, SOD, and PGE2) and decreasing lipid peroxidation byproduct (MDA), which is a major risk factor associated with oxidative stress and increased inflammatory damage in the stomach cells during ulceration (Jabbar 2021, 2022).

**FIGURE 1:**
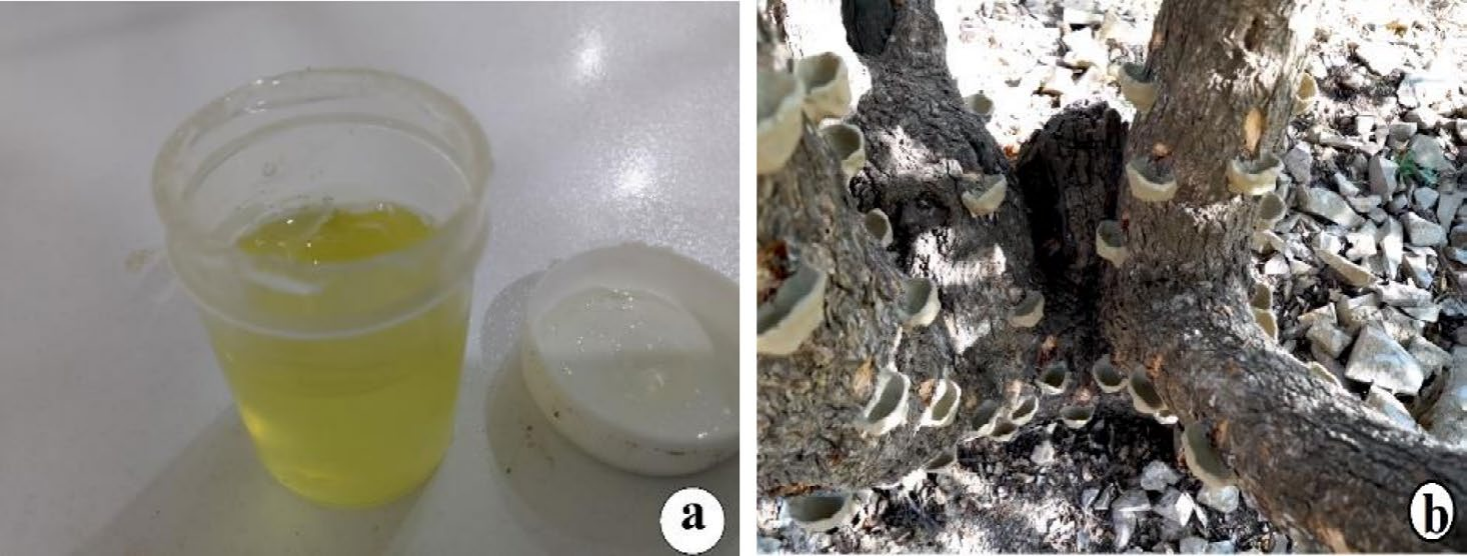
Bene gum (a) and muddy nests as a method of resin collection from *P. atlantica* (b).

*Pistacia atlantica (*Dareben or Dar qezwan*) subsp*. *kurdica Zohary* is a well‐known Kurdish wild flowering plant tree found in the Zagros Mountains of Kurdistan (Sharifi 2014). Dareben (local name) grows naturally all over the Kurdistan region (Haji Omaran, Safeen mountain) belonging to the Anacardiaceae family that exerts an increased amount of resin during the summer season (mostly in August) (Figure 1a), for which several nests of mud are created along the tree trunk before making small cuts for gum exudation (Ben Ahmed et al. 2021) (Figure 1b). The traditional utilization of *P. atlantica* includes a variety of preparation methods (freshly, dissolved in hot water, or cocked with oil) for many human disorders (gastrointestinal disorders including gastric ulcer, gastric acidity, digestive problem, Flatulence, nausea, and diarrhea) (Minaiyan et al. 2015). Phytochemical evaluation of *P. atlantica* gums revealed α‐pinene and β‐pinene as major constituents of their resin, while their essential oils had monoterpenoids (limonene, sabinene, Terpinen‐4‐ol, and elemol) as main chemical constituents. *P. atlantica* resin was found to be the major part that was evaluated for its biological and pharmacological properties. Moreover, phytochemical investigation on five genotypes of *P. atlantica* revealed increased phenolic and flavonoid contents in their resin exudates from plant stems (Hatamnia et al. 2014). *P. atlantica* gums can have numerous biological potentials including antibacterial (Hama Amin et al. 2022), antioxidant (Khiya et al. 2021), enzyme inhibitory (Achili et al. 2020; Benmohamed et al. 2023), anti‐diabetic (Majd et al. 2022), anti‐hyperlipidemic (Hosseini et al. 2020), and anti‐proliferative (Albalawi et al. 2022). The PAG has shown increased inhibitory action against acetylcholinesterase (AChE), cutaneous leishmaniasis (Dolatyabi et al. 2022), anti‐proliferative (MCF‐7) (Pasban‐Aliabadi et al. 2019), and anti‐inflammatory (Benmohamed et al. 2023), alpha‐amylase and alpha‐glucosidase (Raziani et al. 2022).

Oxidative stress as an outcome of imbalance in the formation and elimination of reactive oxygen species (ROS) has been a major risk factor for the initiation of many inflammatory and apoptotic pathways in the gastric cells by decreased immune barriers and the activation of genes associated with antioxidants that contribute majorly to the formation of severe gastric injury (Jabbar et al. 2022b). The pharmacognostic and pharmacological analysis of herbal medicines has increased in recent years to ascertain their therapeutic actions. Despite numerous biological reports on PAG, its traditional utilization as an anti‐ulcer remedy is yet to be explored. Therefore, the present study attempted to investigate the gastric protection effects of PAG on ethanol‐induced gastric ulcers in rats by different histopathological and immunohistochemical assays.

## 2 Methods

### 2.1 Plant Preparation

*P. atlantica* gums were collected from Derbendıˆ Ranya‐Sulaymaniyah, Iraq. The plant species were authenticated by Prof. Abdulla Sardar. The crude resinous gum was dissolved in methanol and dichloromethane solvents in a 4:1 range. After filtration of the mixture using 45 μm pore‐size filter paper, the solvents were evaporated by rotary evaporator at 20°C for 15 min. The obtained amorphous yellow resin, 425 g (85%) was stored in dark bottles for later use (Rajabi et al. 2024). The gum solution was prepared for the in vivo trials by dissolving 100 g in 100 mL of a 10% tween 20 solution.

### 2.2 Acute Toxicity Test

The toxic effects of PAG were evaluated according to the OECD guideline (Guideline 2001). Thirty‐six Sprague Dawley rats (18 males and 18 females) were given by Cihan University‐Erbil, and they were grouped into 3 groups: normal control rats (A) received orally 10% tween 20 by gavage; low (B) and high doses (C)‐treated rats received 2 and 5 g/kg of PAG, respectively (Jabbar 2021). Rats were fastened (free water access) for 1 day before treatments were given. The food was also removed for 3–4 h after supplementation. The animal observation began after dosage delivery and continued at different time intervals for any physiological changes or mortality. After that, rats were ingested. On the last day, food was taken away, and on the 15th day, all rats received anesthesia (ketamine and xylazine) and were sacrificed (Salih et al. 2021). The intracardiac puncture blood samples were biochemically analyzed. The obtained organs (kidney and liver) were histologically examined by using hematoxylin and eosin stains (Al‐Medhtiy et al. 2024).

### 2.3 Experimental Animals

The Sprague Dawley male rats (180–200 g) aged 7–8 weeks were provided by the Animal House Unit, Cihan University‐Erbil. The rats dieted on a normal diet ad libitum and had water for 1 week for adaptation purposes (Jabbar, Mothana, et al. 2023).

### 2.4 Stimulation of Gastric Ulcer

Rats were grouped in widespread mesh‐wired cages to prevent coprophagia. Rats were randomly transferred into 5 cages (6 rats in each). For adaptation purposes, rats were allowed to have a normal diet ad libitum and water for 7 days as an adaptation procedure. One day before the treatment, all rats were fasted (only food) and the drinking water was also removed 2 h before they received the following:

Group A rats received orally 10% tween 20.

Group B ulcer control rats had orally administered 10% tween 20.

Group C rats had an oral dose of 20 mg/kg omeprazole after dissolving in 10% tween 20.

Group D and E rats received 250 and 500 mg/kg of PAG, respectively.

One hour later, group A ingested normal saline, and rats in groups B–E received a single oral dose of 5 mL/kg of 100% alcohol (5 mL/kg) for stomach ulcer induction. After 1 hour, all rats were anesthetized using ketamine and xylazine, sacrificed, and the intracardial blood samples were biochemically evaluated (Jabbar, Mothana, et al. 2023; Jabbar et al. 2022b).

### 2.5 Gross Study

The obtained stomach samples were opened at a, greater curvature and washed several times with a buffer solution. The stomach lesions were noticed as thick red‐line cuts, and gross views were captured using Image J software. The inhibition percentages (I %) were calculated as follows:

$$I\%=\left(\mathrm{UA}\;\text{control}-\mathrm{UA}\;\text{treated}/\mathrm{UA}\;\text{control}\right)\times 100$$

### 2.6 Evaluation of Gastric pH

The obtained stomach contents were tested for hydrogen ion content by a pH meter (mEq/L) (inoLab pH 7310P BNC, Weilheim, Germany) and NaoH 0.1 N (Ibrahim et al. 2022).

### 2.7 Gastric Mucus Contents

The buffer‐washing of stomach samples was performed with phosphate‐buffered saline (PBS) and clean slides were used to examine the gastric mucosal layers. The amount of stomach mucus was measured by electrical balance (Shareef et al. 2022).

### 2.8 Evaluation of Stomach Tissue Homogenates

The gastric tissues were sliced and washed with Phosphate buffer. The gastric tissues were homogenized (1000 g of gastric homogenates spined at 4 °C for 10 min with PBS 10% *w*/*v*) to obtain a cocktail of mammalian protease inhibitors. The separated supernatants were evaluated for the antioxidants (SOD, CAT, PGE2) and malondialdehyde levels. The concentrations of immunohistochemical (Bax, (E‐EL‐R0098) and HSP 70, (E‐EL‐R0479)) in stomach homogenates were determined using an ELISA Kit and Laboratory protocols provided by the producer's company (Elab Science, Wuhan, China) (Zhou et al. 2020).

### 2.9 Histological Analysis

The rats were dissected, and the stomach organs are were obtained from all rat groups. The gastric slices (1–2 cm) were moved into small bottles of formalin (10%) at 25°C for 24 h. Before paraffinization, the tissue slices were treated with different solvents (ethanol and xylene). Finally, slices (5 m) were from paraffin blocks fixed on slides, stained, and viewed using light microscopic (Leica Rotation Microtome) (Abdul‐Aziz Ahmed et al. 2024).

### 2.10 H&E Stain

The gastric tissue slides were colored using histopathological procedures by using Hematoxylin and Eosin stains based on the procedure mentioned previously (Mariod et al. 2023).

### 2.11 Evaluation of Inflammatory Mediators

The obtained blood specimens were centrifuged, and the supernatant was evaluated for the inflammatory cytokine levels using specialized kits purchased from Sigma Aldrich (Merck, Germany) following the producer's instructions (Jabbar, Alamri, et al. 2023).

### 2.12 Statistical Analysis

Data are analyzed using One‐way analysis of variance (ANOVA) and the post hoc test. The graphs were designed by GraphPad Prism (version 9.0). Data are given as Mean ± SEM (*n* = 6) with statistical difference at *p* < 0.05.

## 3 Results

### 3.1 PAG Toxicity

**FIGURE 2:**
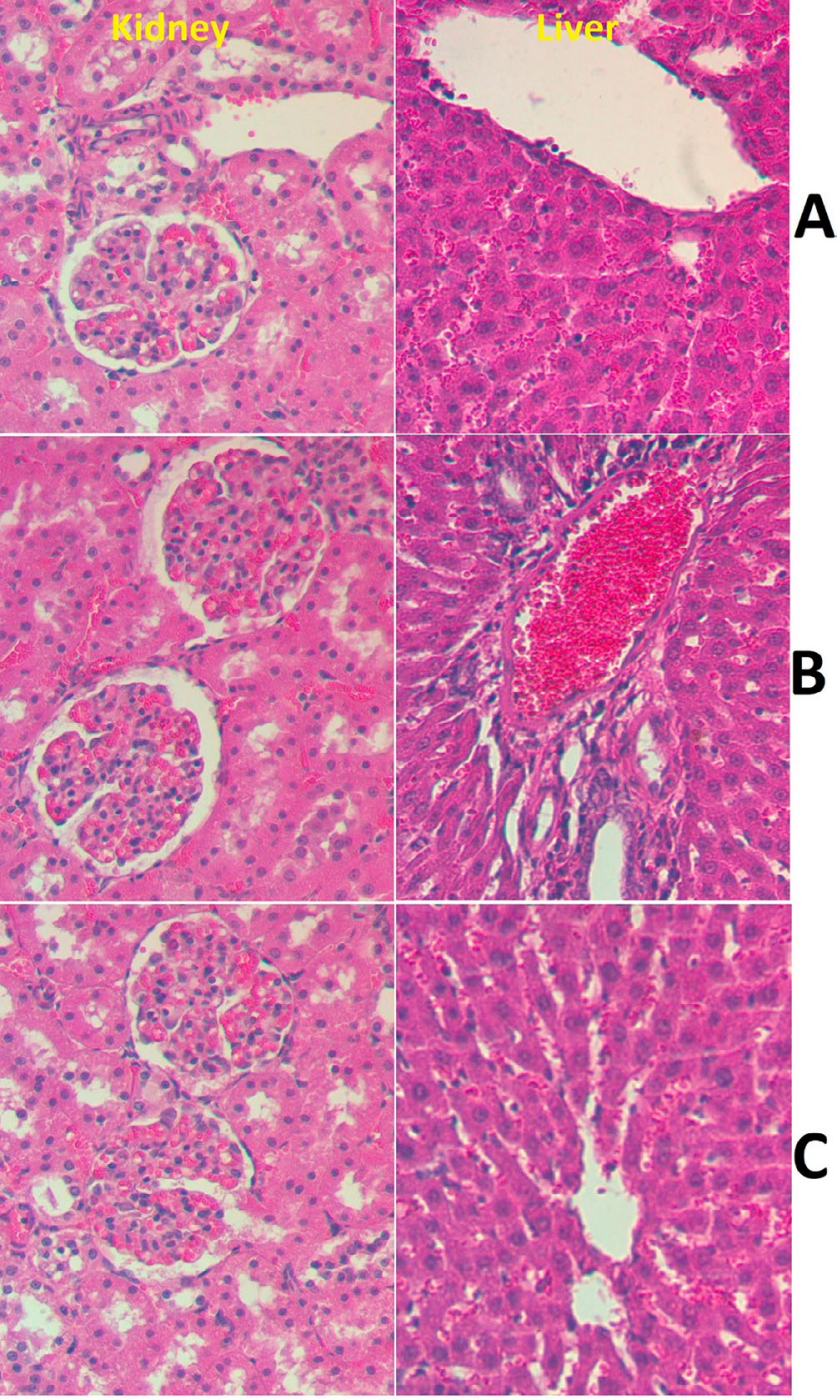
Histological parts of kidney and liver in acute toxicity test. Group (A) received 10% tween 20; (B) 2000 mg/kg PAG‐treated rats; (C) 5000 mg/kg PAG‐treated rats. The PAG supplementation did not cause any structure tissue disruption of dissected liver and kidney organs based on its similar microscopic observation compared to normal control rats (H&E, 40×).

Data analysis showed the non‐toxic effects of PAG supplementation (2 and 5 g/kg dosage) in rats after 2 weeks of supplementation. Rats seemed normally functioning with the absence of physiologic or observable pathological signs. Furthermore, continuous observation showed the absence of any abnormalities in the body weight, food intake, or behavior of 3 experimental groups, with zero death records at the end of the trial. PAG‐treated rats showed no signs of toxicity, convulsion, tremor, biting, eye color change, depilation, or diarrhea. The histological analysis revealed similar kidney and liver tissue composition between normal control and PAG‐supplemented rats (Figure 2). The biochemical estimation showed non‐significant alteration in the liver (Table 1) and kidney (Table 2) parameters of PAG‐supplemented rats compared to normal controls. The outcomes denote that the toxicity dosage of PAG would be higher than 5 g/kg.

**TABLE 1:** Influence of PAG on some liver function tests of rats in toxicity tests.

Groups	Albumin g/L	Total bilirubin µmol/L	Alkaline phosphatase U/L	Alanine aminotransferase U/L	G‐glutamyl transferase U/L
A	32.2 ± 1.8	< 2	166.2 ± 2.4	78.32 ± 11.8	0.07 ± 0.5
B	33.1 ± 2.0	< 2	162.71 ± 3.2	80.12 ± 14.1	0.08 ± 0.4
C	35.3 ± 3.0	< 2	168.30 ± 3.5	83.56 ± 13.2	0.09 ± 0.5

*Note:* A, vehicle rats received orally 10% tween 20; B and C, were rats received 2000 and 5000 mg/kg of PAG, respectively.

**TABLE 2:** Effects of PAG on some kidney functional tests of rats in an acute toxicity trial.

Groups	Sodium mmol/L	Potassium mmol/L	Chloride mmol/L	Carbon dioxide mmol/L	Anion gap mmol/L	Urea mmol/L	Creatinine mmol/L
A	139.2 ± 5.2	4.74 ± 1.6	101.5 ± 3.6	34.30 ± 1.9	3.4 ± 0.8	6.80 ± 1.4	21.20 ± 3.2
B	142.5 ± 4.2	5.23 ± 1.8	105.08 ± 3.2	35.22 ± 1.3	4.0 ± 0.7	6.10 ± 1.8	24.10 ± 3.0
C	141.3 ± 5.1	5.45 ± 2.1	102.2 ± 4.3	36.16 ± 2.1	3.1 ± 0.4	7.12 ± 2.3	2.6 ± 4.5

*Note:* Values are shown as Mean ± SEM (*n* = 6). Values are considered non‐significant at *p* < 0.05. G1 and G2 were rats that received 2500 and 5000 mg/kg of PAG, respectively.

### 3.2 Gastroprotective Actions of PAG in an Ulcerative Animal Model

#### 3.2.1 Effect of PAG on Gross Views

**FIGURE 3:**
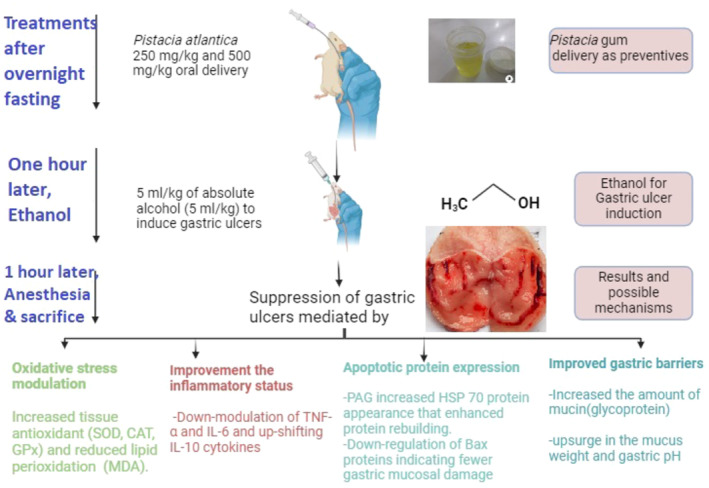
Gastroprotective procedure for PAG and its depicted molecular mechanisms.

**FIGURE 4:**
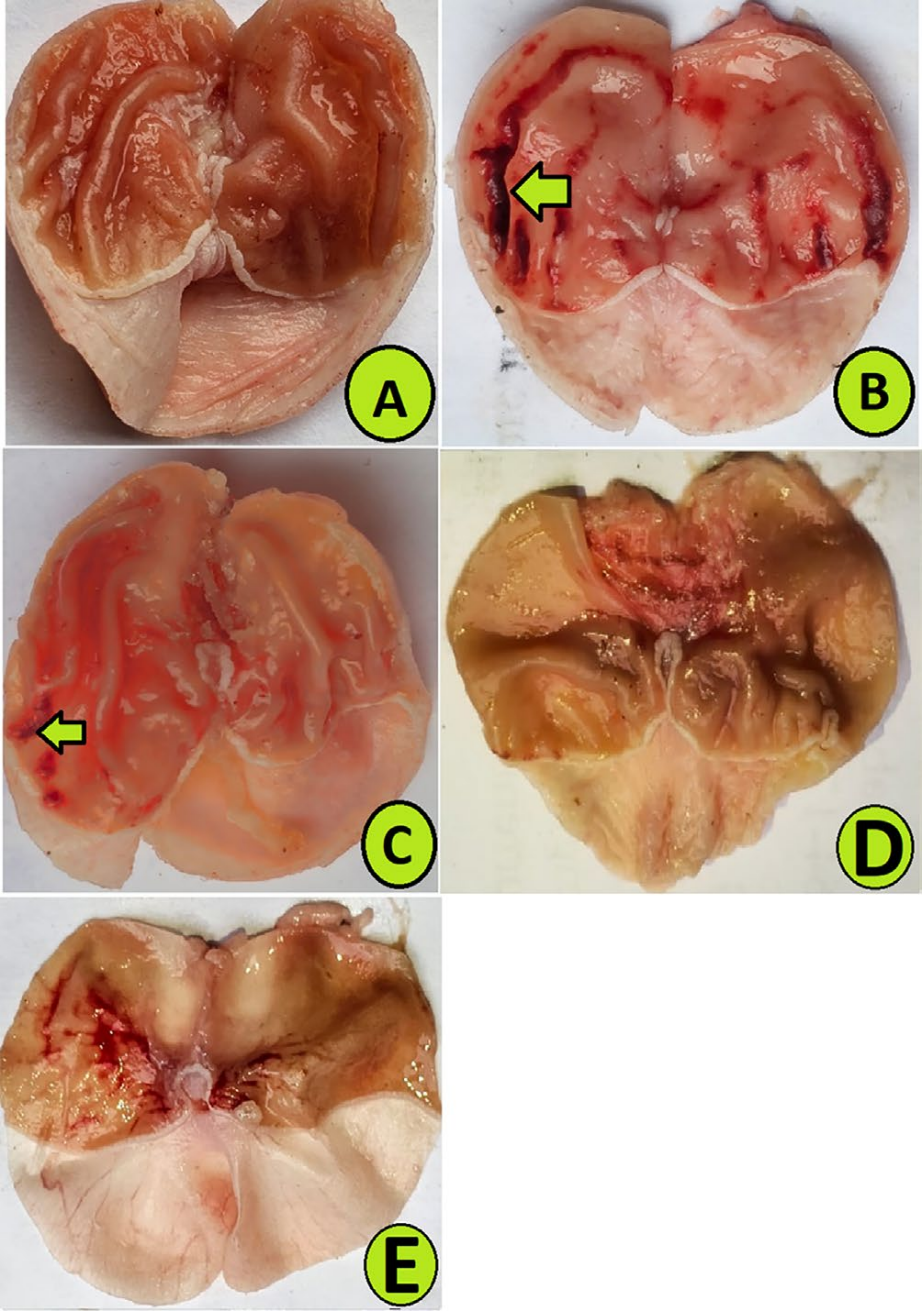
Shows gross stomach views of rats ingested with different treatments. (A) Vehicle rats received orally 10% tween 20; (B) 10% tween 20 + ethanol; (C) 20 mg/kg omeprazole + ethanol; (D) 250 mg/kg PAG + ethanol; (E) 500 mg/kg + ethanol. The arrows indicate the mucosal lesions in differently treated rats.

The stomach gross views showed that rats in group A had usual gastric mucosal layers. In contrast, rats that received only ethanol (B) had severe mucosal lesions in their stomach with numerous lesion areas on the gastric mucosa. Rats in group C (reference medicine) had the lowest stomach injury in their mucosal layers based on gross observation, which could be attributed to various molecular mechanisms (Figure 3). Rats ingested 250 mg/kg of PAG (D) showed notably lower gastric lesions than those of group B (ulcer rats) (Figure 4). Rats who received a high dose of PAG (500 mg/kg) (E) showed flat epithelial surfaces of gastric tissues with notably reduced gastric mucosal damage compared to that of group B and D rats.

#### 3.2.2 PAG Effects on Mucus Levels

As expected, normal control rats (A) revealed efficient mucus content with a lack of any lesion indications. Ulcer controls (B) showed reduced mucus secretions and increased stomach lesions compared to treated rats, which also means lower glycoproteins, glycolipids, glycogen, and mucin in stomach tissues that consequently led to severe mucosal damage and lesion formation. Rats treated with omeprazole (C) had similar mucus content as normal controls and reduced stomach lesions. PAG‐treated rats (D and E) resisted ethanol‐mediated gastric lesions, indicated by moderate mucus content (higher polysaccharides) and lower stomach ulcers compared to ulcer controls (Table 3).

**TABLE 3:** Influence of PAG ingestion on stomach profiles.

Animal groups	Mucus weight (g)	pH	Ulcer area (mm)^2^	Inhibition
A	2.17 ± 0.22^a^	6.11 ± 0.067^a^	—	—
B	0.61 ± 0.05^e^	3.70 ± 0.20^e^	593.16 ± 30.80^d^	—
C	1.84 ± 0.40^b^	5.78 ± 0.22^b^	110.33 ± 7.0^a^	81.39%
D	1.36 ± 0.82^d^	4.56 ± 0.27^d^	211.83 ± 47.43^c^	64.28%
E	1.62 ± 0.11^c^	4.97 ± 0.13^c^	145.66 ± 16.58^b^	75.44%

*Note:* Values shown as Mean ± SEM (*n* = 6). were labeled by similar letters on the same column. Group A rats received orally 10% tween 20; group B rats had 10% tween 20 + ethanol; group C had received 20 mg/kg omeprazole+ ethanol; group D rats received 250 mg/kg PAG + ethanol; E, 500 mg/kg PAG + ethanol. Labeled values by similar letters on the same column are considered non‐significant values (*p* < 0.05).

#### 3.2.3 PAG Effects on the Gastric pH

Rats treated with ethanol alone (B) had the lowest gastric pH compared to experimental and normal control rats. Rats ingested with omeprazole (C) had a similar gastric pH as normal control rats. Gastric pH was gradually increased in a dose‐related manner in PAG‐treated rats. Rats ingested 250 mg/kg PAG showed significantly higher gastric pH compared to ulcer controls. While rats that had 500 mg/kg had better gastric pH recovery compared to ulcer controls and 250 mg/kg PAG‐treated rats, as the rats treated with 500 mg/kg PAG. Overall, PAG treatment significantly preserved gastric pH in ethanol‐induced stomach ulcer rats (Table 3).

#### 3.2.4 H&E Stain

**FIGURE 5:**
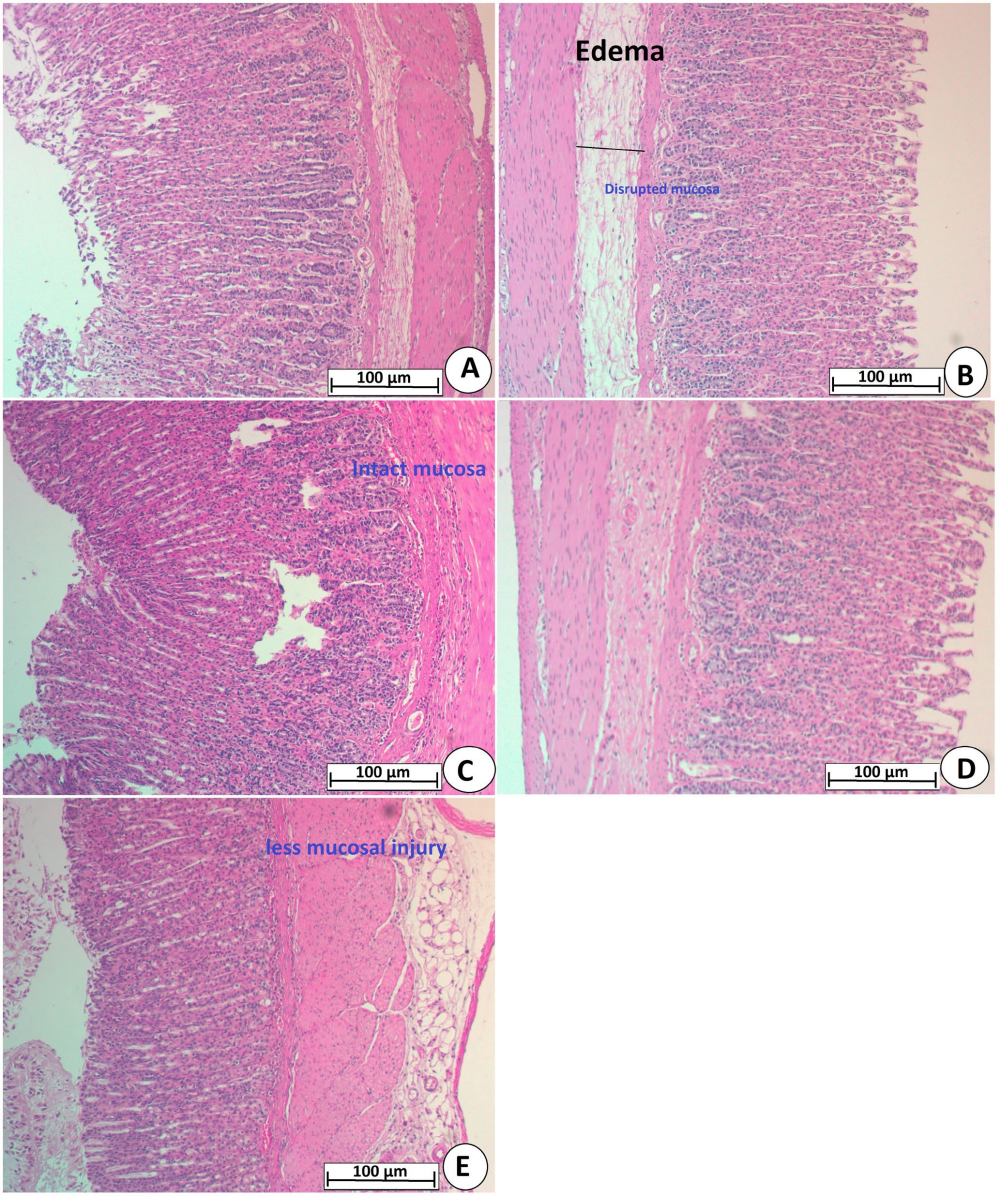
Effect of PAG on ethanol‐mediated gastric ulcer in rats. Gastric tissues stained with hematoxylin and Eosin stain revealed that Normal control rats (A), had the normal microscopic structure of gastric mucosa; Ulcer control rats (B), had numerous structural damages in the mucosa presented by edema and leucocyte infiltration; ethanol +20 mg/kg Omeprazole group (C), had a mild gastric injury; ethanol + PAG 250 mg/kg (D), showed moderate penetration of gastric mucosa; ethanol + PAG 500 mg/kg (E), showed fewer gastric tissue disruptions (H&E stain, 20×). Black line, mucosal disruption.

Rats receiving only ethanol showed clear severe gastric tissue penetrations, ulcerations, and numerous lesions in the gastric mucosa with leukocyte infiltrations. Experimental rats treated with omeprazole or PAG (250 or 500 mg/kg) had significantly less gastric epithelial damage, with lesser ulcer area and edema in the submucosal parts (Figure 5).

#### 3.2.5 Effect of PAG on the HSP 70 and Bax Expressions

**FIGURE 6:**
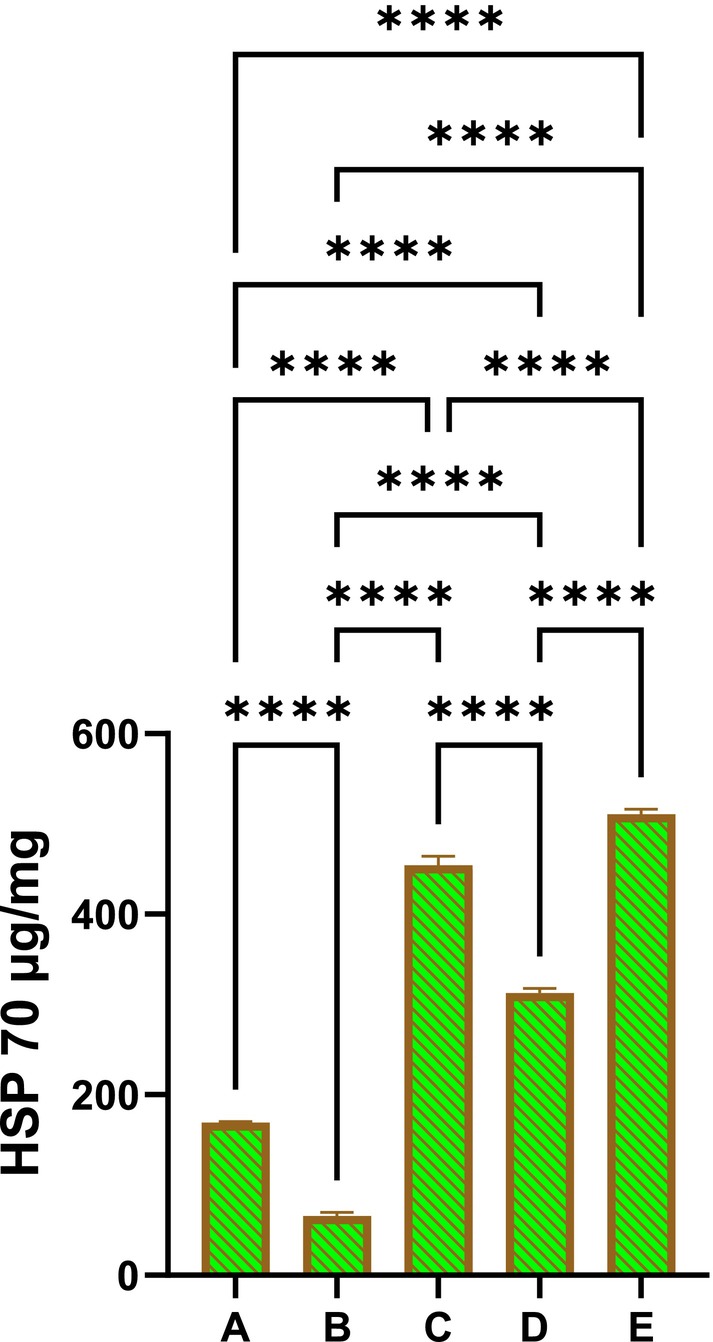
Effects of *PAG* on the expression of HSP 70 proteins in stomach tissues of different treated rats. Normal control rats showed reduced HSP 70 protein intensity in their stomach mucosa (A), enhancing increased apoptotic actions that led to severe gastric mucosal injury and edema with leukocytes in their stomach mucosa (B). Omeprazole‐treated rats experienced mild stomach injury and the highest HSP 70 protein intensity in their gastric mucosa (C). Rats received 250 mg/kg had moderate stomach injury and up‐regulated HSP 70 proteins in their stomach mucosa compared to ulcer control rats (D). Rats who received a higher dose (500 mg/kg) of PAG exhibited mild gastric injury and significantly increased HSP 70 appearances in their stomach tissue homogenates limited to ethanol‐mediated apoptotic stomach damage (E).

The immunohistochemical examination of stomach tissue homogenates stained with immunostains revealed different expressions of HSP 70 and Bax tissue proteins. The normal control rats (Figure 6A) showed minimal expression of HSP 70 proteins in their stomach tissues, indicating the absence of apoptotic actions in their cells. The ulcer control rats that received only ethanol + 10% tween 20 showed reduced HSP 70 intensity in their stomach tissues (Figure 6B). Rats pre‐treated with Omeprazole (20 mg/kg) or PAG (250 or 500 mg/kg) showed significantly increased HSP 70 protein intensity in their stomach tissues, indicating a clear anti‐apoptotic the action of this protein that limits toxic damage and cellular disruptions induced by absolute ethanol oral delivery (Figure 6C–E).

**FIGURE 7:**
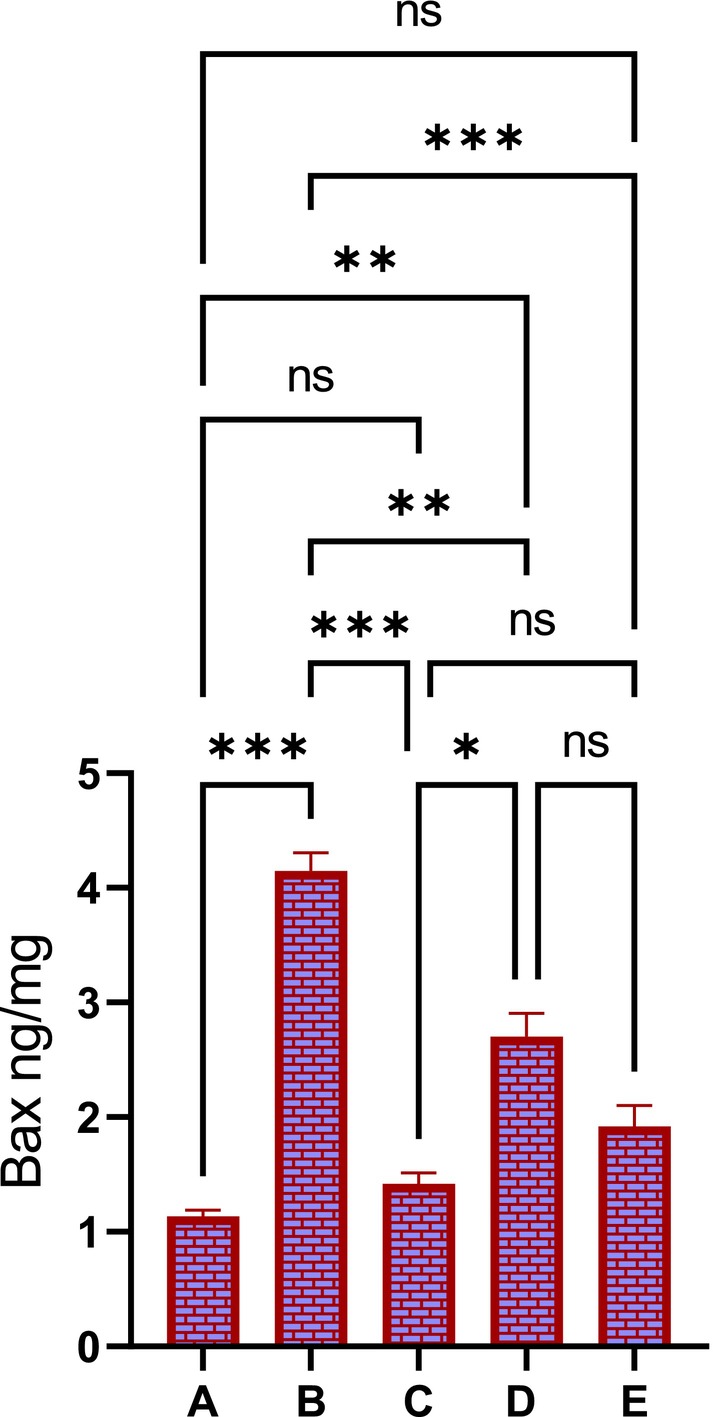
Effects of PAG on the Bax protein intensity in stomach tissues of different treated rats. Normal control rats showed very reduced Bax protein intensity in their stomach mucosa, almost denoting no apoptotic actions (A). Ulcer control rats exhibited increased Bax protein intensity in their stomach mucosa, denoting heavy ethanol‐mediated tissue damage and enhanced apoptotic actions (B). Omeprazole‐treated rats showed mild stomach injury and reduced Bax protein intensity in their gastric mucosa (C). Rats receiving 250 mg/kg had moderate mucosal damage and decreased Bax protein in their stomach tissues compared to ulcer control rats (D). Rats pre‐treated with 500 mg/kg of PAG showed mild gastric tissue injury and significantly lower Bax protein expression in their stomach tissues compared to the ulcer control rats. These were found comparable and non‐significantly varied compared to the reference drug‐treated groups but were significantly different from those of the ulcer control group (E).

The Bax protein expressions were significantly varied between different treated rats, which were approved by various intensities of Bax protein in the stomach tissues. As expected, normal controls had almost an absence of apoptotic actions represented by reduced Bax protein appearance in their stomach tissues. However, ulcer controls revealed noticeably increased Bax protein intensity in their stomach tissues, denoting severe cell injury and elevated apoptotic actions as a result of ethanol‐mediated toxic damage. Moreover, Rats pre‐treated with omeprazole (20 mg/kg) or PAG (250 or 500 mg/kg) had significantly lower Bax protein expressions compared to those of ulcer control rats (Figure 7).

#### 3.2.6 PAG Effects on Gastric Antioxidants

**FIGURE 8:**
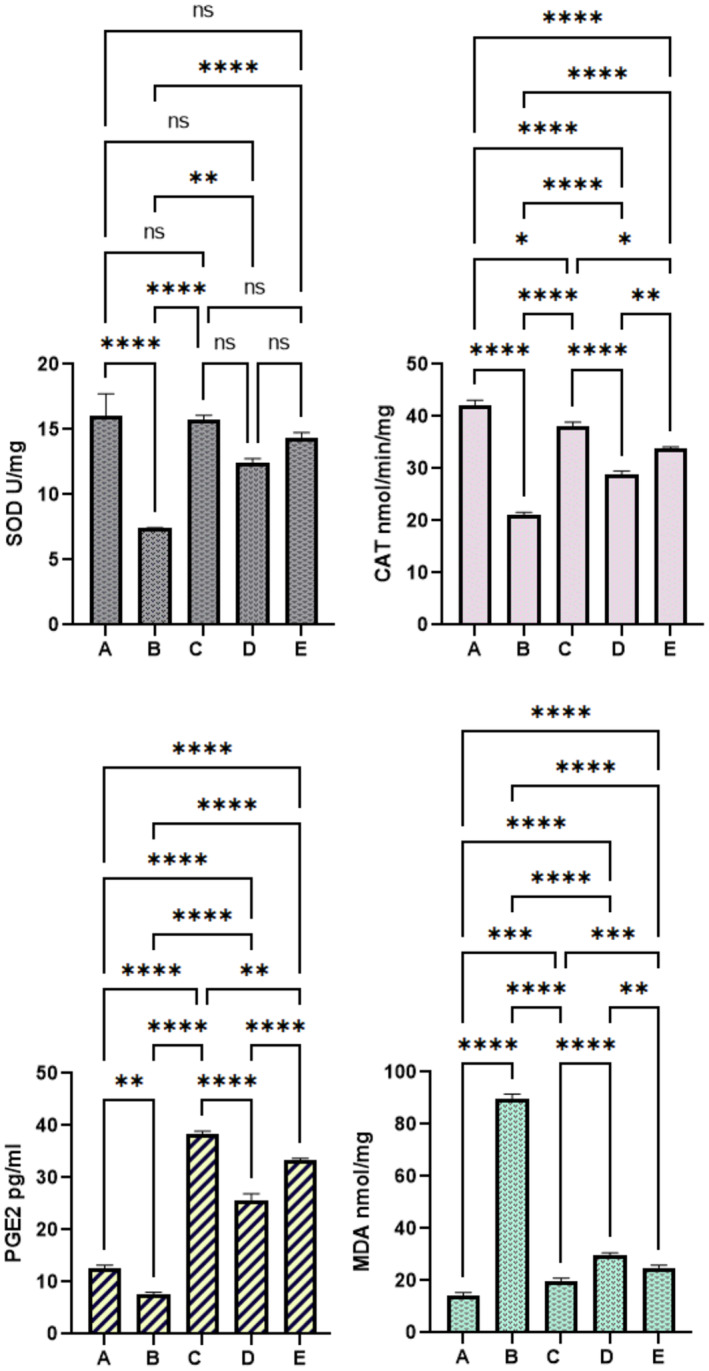
Effect of PAG on antioxidants and MDA levels in rats. (A) Vehicle rats received orally 10% tween 20; (B) 10% tween 20 + ethanol; (C) 20 mg/kg omeprazole + ethanol; (D) 250 mg/kg PAG + ethanol; (E) 500 mg/kg PAG + ethanol.

Biochemical analysis showed that group A exhibited normal antioxidant actions and the lowest lipid peroxidation (MDA) values compared to all experimental rats. Ulcer control rats (B) revealed notably fewer antioxidant (SOD, CAT, and PGE2) activities than those of the reference drug (C) or PAG‐treated rats (D and E) (Figure 8). Rats treated with reference medicine had almost the same gastric antioxidant values as normal control rats. PAG treatment caused a significant up‐rising of gastric antioxidant levels compared to ulcer controls (B). Rats that ingested 500 mg/kg PAG had higher SOD (14.34 U/mg) and CAT (33.83 nmol/min/mg) values than those (12.47 and 28.88 ); (7.40 and 22) of 250 mg/kg PAG and ulcerated rats, respectively. MDA values were statistically lower in 500 mg/kg PAG‐treated rats (24.97 ± 4.24) than those (89.62 ± 6.2) and (29.83 ± 5.8) of the B and D groups, respectively. Furthermore, PAG treatment caused a significant increase in PGE2 values, which were statistically higher than in the ulcer control (Figure 8).

#### 3.2.7 Influence of PAG on the Cytokines

**FIGURE 9:**
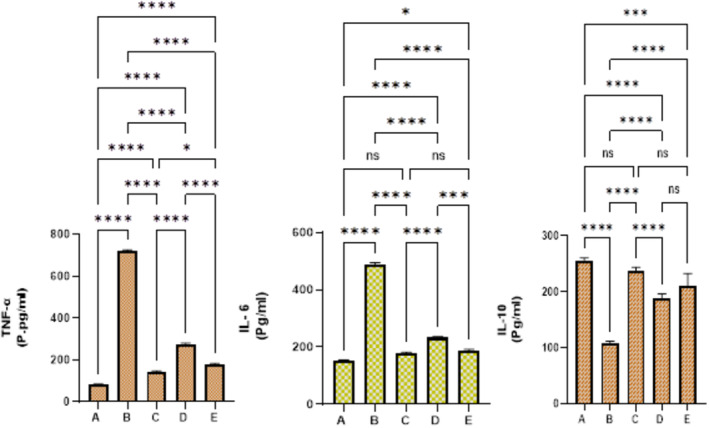
Effects of PAG on the inflammatory cytokines in experimental rats. (A) Vehicle rats received orally 10% tween 20; (B) 10% tween 20 + ethanol; (C) 20 mg/kg omeprazole + ethanol; (D) 250 mg/kg PAG + ethanol; (E) 500 mg/kg PAG + ethanol. Values are shown as means ± SEM (*n* = 6). The present oral ingestion of PAG caused significant immunomodulatory action represented by reduced TNF‐α and IL‐6 levels and increased IL‐10 levels compared to ulcer controls.

The current data analysis revealed an increased immunomodulatory effect of PAG in alcohol‐induced ulceration in rats. As expected, vehicle rats revealed reduced serum inflammatory TNF‐α, IL‐6 values, and elevated IL‐10 cytokines. The ulcer control rats showed the highest inflammation rate represented by higher TNF‐α, IL‐6 cytokines, and lower IL‐10 levels compared to all groups. The reference rats showed a better serum inflammatory profile (lower TNF‐α, and IL‐6 levels and increased IL‐10 cytokines) than the ulcer controls. The current plant supplementation (250 or 500 mg/kg) by PAG to rats results in a significant improvement in the immunologic status shown by reduced TNF‐α and IL‐6 levels and increased IL‐10 levels, subsequently accelerating the recovery process of gastric lesions caused by alcoholic oral ingestion (Figure 9).

## 4 Discussion

The acute toxicity test is a well‐known safety measurement to estimate an interesting herbal product's lethal dosage (LD_50_) (Jabbar, Alamri, et al. 2023). The current acute toxicity experiment found non‐toxic effects nor death in rats ingested with 2 and 5 g/kg PAG, indicating a higher than 5000 mg/kg of PAG as the toxic dosage of this plant spp. Similarly, the safety of 25, 50, and 100 mg/kg of PAG was shown in a rat trial (Tanideh et al. 2021). Accordingly, 2 g/kg ingestion of essential oils from PAG was safe without any observable toxic signs in rats (Memariani et al. 2017).

The pathophysiology of gastric ulcers is still not fully determined; however, scientists have declared that most gastric ulcers result from impaired homeostasis between tissue disruption factors and the gastric mucosal defense system (Antioxidant enzymes) (Jabbar 2022). Gastric barriers and defensive pathways could be weakened by chemicals (ethanol) through the formation of lesions in the gastric epithelial layers and increasing vascular permeability (edema). Alcohol can damage the gastric mucous layer, leading to necrotic lesions by several pathways, decreasing gastric motility, lowering bicarbonate secretion, and reducing the production of gastric mucus (Fu et al. 2022). In the current research, the ethanol oral gavage in rats significantly lowered the thickness of gastric mucosa, declined mucus secretion, and down‐regulated antioxidant enzymes detected in gastric tissue homogenates. Eventually, ethanol caused the increased formation of ulcer area, induced acid secretion, and interruption of the gastric epithelium along with the production of ulcers and hemorrhage among damaged gastric glands. Similarly, researchers have reported ethanol efficacy in the induction of peptic ulcers in various rat trials (Jabbar, Mothana, et al. 2023; Ahmed et al. 2024).

The PAG treatment significantly protected the gastric epithelium from absolute ethanol‐mediated tissue disruption. PAG treatment increased gastric surface area and flattened mucous linings in the gastric epithelia, and thus rats had lower gastric injury than ulcer control rats. Accordingly, researchers revealed the anti‐ulcer potentials of several herbal medicines, and they have linked this bioactivity with their natural chemical contents that can exhibit anti‐radical quenching action, thereby lowering oxidative stress related to stomach cell injury (Shin et al. 2021). Moreover, researchers have reported significant gastroprotective potentials of essential oil of PAG (25, 50, and 100 mg/kg) against ethanol‐mediated gastric lesions in rats, which were linked with its inhibitory action on the amount and the acidity of gastric secretions, due to its phytochemical (α‐pinene, citral, myrtenol, carveol, epoxypinene, and β‐pinene) potentials in blocking the gastric receptors in the parietal cell (muscarinic receptors (M3), H2—histamine receptor and CCKb—gastrin receptor) (Memariani et al. 2017). Because activation of these receptors (by ethanol) leads to the up‐regulation of gastrin regulators, histamine release, and blood supply for gastric mucosa, thereby facilitating further gastric secretion and reduction of protective factors (Ali et al. 2017).

The anti‐ulcer action of PAG against ethanol‐induced ulcers can be correlated with its potential to decrease cell permeability of gastric submucosa layers, induction of gastric juice formation, and leukocyte infiltration (up‐rising stomach pH), thereby lowering rates of inflammation. Similarly, scientists have correlated the anti‐inflammatory action of volatile oils obtained from PAG (100, 200, and 400 μL/kg) with its chemical content (phenolics, terpenoids, and flavonoids) that lowered the rate of colitis and tissue myeloperoxidase action in acetic acid (4%)‐induced colitis in rats (Minaiyan et al. 2015). Similarly, oleoresin essential oil (25, 50, and 100 mg/kg orally) from *Pistacia atlantica* ameliorated the gastric ulceration model, which was mainly linked with its GC/MS‐detected phytochemicals, including α‐pinene as the most abundant chemical (Memariani et al. 2017).

The immunohistochemical proteins, which are under the effect of ethanol‐induced oxidative stress, can have modulatory effects on the apoptotic process and thereby further stomach tissue injury. Ethanol oral delivery can create a severe oxidative gastric tissue injury, which will cause significant down‐regulation of HSP 70 proteins and up‐shift in the Bax protein appearance in tissue homogenates. As a major gastric epithelium defense component, HSP 70 minimizes oxidative stress‐mediated cell damage and prevents refolding or aggregation of partially denaturized proteins. HSP 70 proteins can be found in the mitochondria, nucleus, cytoplasm, cell membrane, and extracellular space (Jabbar, Mothana, et al. 2023). Its expression can be seen in regions with cellular stress, which will reduce further tissue damage by weakening ROS formation and enhancing the process involved in cellular recovery. In the case of gastric tissue injury, HSP 70 proteins' defensive role includes limitation of oxidative stress damage and promotion of normal protein rebuilding by preventing refolding and then elimination of damaged cellular proteins. As a major pro‐apoptotic protein and a member of the Bcl‐2 family, Bax protein can have a significant modulatory effect on apoptotic actions during cellular injuries. Absolute ethanol oral delivery can create a toxic environment that favors oxidative stress‐mediated stomach injury and increased apoptotic process via up‐regulation of pro‐apoptotic proteins (Bax); in such cases, Bax protein decreases the anti‐apoptotic bodies from B cell lymphoma‐2 (BCL‐2) family (Almaimani et al. 2024). In the present study, ethanol oral ingestion in rats caused significantly decreased HSP 70 proteins and increased Bax protein appearance in their stomach epithelium as seen in ulcer controls. Meanwhile, PAG supplementation (250 and 500 mg/kg) restored these changes induced by ethanol, denoted by higher HSP 70 protein and lower Bax protein appearance in their gastric tissue homogenates compared to ulcer controls. Our data are consistent with the previous studies detailing the modulatory potentials of *Pistacia atlantica* species on the pro‐apoptotic and anti‐apoptotic proteins that limit oxidative stress‐mediated cell injury. Such biological potential of these plants has been mainly linked to their phytochemical contents (flavonoids, phenolic, and saponins) (Albalawi et al. 2022; Saeedi et al. 2024). Similarly, several scientists have shown PAG efficacy in the augmentation of apoptotic proteins represented by increased Bax, reduced Bcl‐2 proteins, and provoking actions on caspase‐9 and ‐3 in numerous cancer cell types, which were mainly linked with its polyphenol chemical constituents of PAG (phenolic acid and flavonoids) (Rahman 2018).

Oxidative stress is a cellular phenomenon that occurs as an outcome of an imbalance between antioxidant production and ROS elimination. Oxidative stress can have a major role in the initiation of many human diseases, which occurs through major mechanisms. The first signaling pathway involves the formation of ROS molecules including •OH, ONOO− and HOCl—that will immediately oxidize large cellular compartments such as proteins, enzymes, membrane lipids, and nucleic acids, consequently leading to cellular dysfunction and cell death. The second pathway of oxidative stress‐related cell damage involves aberrant redox cell signaling, in which the oxidant molecules mainly H_2_O_2_ produced during normal cell functioning can act as a secondary cell signaling molecule (Joshua et al. 2022). During oxidative stress, abnormally increased production of H_2_O_2_ provokes a redox signaling pathway that worsens the disease condition. The incidence of both oxidative stress pathways is possible in a single disease, as in the case of diabetes, where glycation byproducts lead to significant activation of stress signaling mechanisms, consequently increasing diabetic complications (Forman and Zhang 2021). Furthermore, up‐regulation of H_2_O_2_ and iron release from damaged cellular proteins during oxidative stress can occur by O_2_•− and ONOO−, which lead to significantly increased production of lipid peroxidation molecules including 4‐hydroxy‐2‐nonenal (HNE), which enhances the aberrant cellular pathways (Sahoo et al. 2023). The present results revealed increased antioxidant actions of notable PAG, which could be through its phytochemical potentials in the regulation of the aforementioned oxidative‐stress pathways. Accordingly, scientists have reported the anti‐ulcer action of PAG, which was linked to its significant potential in lowering expression of p‐NF‐κB, p65, and p‐IKKα/β and up‐regulating IκB‐α, subsequently reducing inflammatory and apoptotic actions (Khoramdareh et al. 2022).

The present data showed noticeable antioxidant potentials of PAG represented by up‐regulation of SOD, CAT, and PGE2 and reduced MDA levels in the gastric homogenates of rats ingesting ethanol as gastric ulcer inducers. Furthermore, data results reveal significant anti‐inflammatory potentials of PAG shown by increased regulation of PGE2 levels in ethanol‐induced gastric ulcerative rats. Similarly, researchers have shown the antioxidant and inflammatory potentials of PAG in different study trials (Bagheri et al. 2019; Bahrami et al. 2024) experiments, which were mainly due to its enriched chemical contents (phenolic and flavonoids) (Hasheminya and Dehghannya 2020). Therefore, the anti‐ulcer bioactivity of PAG may be due to its quenching free radical actions, thereby lowering oxidative stress damage. Eventually, these will lead to fewer formations of ulcer areas, higher secretion of gastric mucus, increased stomach pH (enhanced gastric repair), and improved anti‐inflammatory defense mechanisms.

The modulation of the pro‐inflammatory cytokines in the stomach tissues is considered an effective preventive method for lowering further stomach damage. The inflammatory process can also be initiated during gastric ulcers by the catalytic mechanism of two basic inflammatory factors: inducible nitric oxide synthetase (iNOS) and cyclooxygenase‐2 (COX‐2), in which the elevated appearance of iNOS protein was associated with enlarged gastric ulcers, and its level was decreased during the final healing stages. The COX‐2 enzyme was found in increased amounts during ethanol‐induced gastric ulcers mainly due to protein up‐regulation (inflammation) because this enzyme is involved in the tissue remodeling process epithelialization of gastric mucosa, maturation of granulation tissue, and regeneration of extracellular matrix (Akanda et al. 2018). In the present ulcer trial, ethanol ingestion caused up‐regulation of pro‐inflammatory mediators and reduced IL‐10 cytokine release, as seen in ulcer controls. Meanwhile, PAG supplementation improved ethanol‐mediated inflammation, denoted by lower serum TNF‐α and IL‐6 and increased IL‐10 serum contents. Accordingly, researchers have shown significant anti‐inflammatory and immunomodulatory (TGF‐β, INF‐γ, IL‐4, IL‐5, IL‐10, and IL‐17) effects of aqueous extract (100, 200, and 400 mg/kg) of PAG in dexamethasone (1 mg/kg; intraperitoneal)‐induced asthmatic rats (Bagheri et al. 2019). Accordingly, *PAG* resin (400 mg/kg) + honey (400 mg/kg) caused significant inhibition of the inflammation of the bowel and colonic ulcer incidence in acetic acid‐induced colitis in rats, which were represented by reduced serum inflammatory mediators (IL‐6 and TNF‐α cytokines) (Gharazi et al. 2021).

## 5 Conclusion

The current gastroprotective evaluation revealed significant ameliorative action of PAG against ethanol‐induced ulcer rats through various histopathological assays. Data outcomes exposed significant anti‐ulcer efficacy of PAG mediated through the increasinggastric mucus secretion and gastric endogenous antioxidants. PAG treatment caused up‐regulated HSP 70 and decreased Bax protein expressions. Moreover, PAG supplementation increased tissue antioxidants (SOD, CAT, and PGE2) and significantly reduced MDA levels in the gastric tissue homogenates. These bioactivities could be correlated with its phytochemical potentials (flavonoids, phenolic, and saponins) in quenching free radicals and ROS suppression by provoking genes that encode for the antioxidant enzymes via Nrf2 mechanisms, thereby reducing oxidative stress‐related disorders (gastric lesions and stomach ulcers). PAG supplementation caused significant reduction of the inflammatory mediators (TNF‐α and IL‐6 cytokines) while up‐regulating IL‐10 formation. The current outcome is scientific evidence toward further examination of the molecular identifications underlying its biological potentials. The current study faced many challenges and limitations, including a small animal house and availability of rats, poor facilities, and lack of specialized instruments; therefore, future experiments on a larger scale may provide stronger outcomes on PAG bio‐potentials.

## Author Contributions

**Talal Salem Al‐Qaisi:** resources (equal), software (equal), supervision (equal), validation (equal). **Ahmed A. J. Jabbar:** conceptualization (equal), data curation (equal), formal analysis (equal), visualization (equal), writing – original draft (equal), writing – review and editing (equal). **Mohammed M. Hussein M. Raouf:** data curation (equal), formal analysis (equal), investigation (equal), software (equal), supervision (equal), validation (equal). **Najat Jabbar Ahmed Berwary:** methodology (equal), resources (equal), software (equal), validation (equal). **Qosay Al‐Balas:** data curation (equal), formal analysis (equal), resources (equal), software (equal), supervision (equal), validation (equal), visualization (equal). **Parween Abdul‐Samad Ismail:** data curation (equal), formal analysis (equal), resources (equal), software (equal), supervision (equal), validation (equal), visualization (equal). **Muzhda Haydar Saber:** data curation (equal), formal analysis (equal), investigation (equal), software (equal), supervision (equal), validation (equal), visualization (equal). **Ramzi A. Mothana:** data curation (equal), formal analysis (equal), software (equal), funding (equal), validation (equal), visualization (equal). **Abdullah R. Alanzi:** data curation (equal), formal analysis (equal), investigation (equal), resources (equal), software (equal), validation (equal). **Mahmood Ameen Abdulla:** conceptualization (equal), investigation (equal), methodology (equal), visualization (equal). **Rawaz Rizgar Hassan:** data curation (equal), formal analysis (equal), investigation (equal), software (equal), supervision (equal), validation (equal). **Musher Ismael Saleh:** conceptualization (equal), data curation (equal), formal analysis (equal), funding acquisition (equal), resources (equal), software (equal). **Sidgi Hasson:** data curation (equal), formal analysis (equal), resources (equal), software (equal), validation (equal), visualization (equal).

## Ethics Statement

The study was conducted in compliance with the ARRIVE guidelines (Sirois 2022). The animal experiment was approved by the Tish University Ethical Committee, Erbil, Iraq (BIO. 234, 11/12/2024/M.A.A.).

## Conflicts of Interest

The authors declare no conflicts of interest.

## Data Availability

Further details will be available on request.
